# Stable Cas9 expression regulates cell growth by facilitating mTORC2 activation

**DOI:** 10.1093/nar/gkaf965

**Published:** 2025-09-29

**Authors:** Le Yu, Yi Jin, Jianfeng Chen, Zhichuan Zhu, Siyuan Su, Emily M Wilkerson, Joanna Gongora, Erica W Cloer, Michael B Major, Pengda Liu

**Affiliations:** Lineberger Comprehensive Cancer Center, The University of North Carolina at Chapel Hill, Chapel Hill, NC 27599, United States; Department of Biochemistry and Biophysics, The University of North Carolina at Chapel Hill, Chapel Hill, NC 27599, United States; Lineberger Comprehensive Cancer Center, The University of North Carolina at Chapel Hill, Chapel Hill, NC 27599, United States; Department of Biochemistry and Biophysics, The University of North Carolina at Chapel Hill, Chapel Hill, NC 27599, United States; Lineberger Comprehensive Cancer Center, The University of North Carolina at Chapel Hill, Chapel Hill, NC 27599, United States; Department of Biochemistry and Biophysics, The University of North Carolina at Chapel Hill, Chapel Hill, NC 27599, United States; Lineberger Comprehensive Cancer Center, The University of North Carolina at Chapel Hill, Chapel Hill, NC 27599, United States; Department of Biochemistry and Biophysics, The University of North Carolina at Chapel Hill, Chapel Hill, NC 27599, United States; Lineberger Comprehensive Cancer Center, The University of North Carolina at Chapel Hill, Chapel Hill, NC 27599, United States; Department of Biochemistry and Biophysics, The University of North Carolina at Chapel Hill, Chapel Hill, NC 27599, United States; Department of Cell Biology and Physiology, Washington University, St. Louis, MO 63110, United States; Department of Cell Biology and Physiology, Washington University, St. Louis, MO 63110, United States; Lineberger Comprehensive Cancer Center, The University of North Carolina at Chapel Hill, Chapel Hill, NC 27599, United States; Department of Cell Biology and Physiology, Washington University, St. Louis, MO 63110, United States; Lineberger Comprehensive Cancer Center, The University of North Carolina at Chapel Hill, Chapel Hill, NC 27599, United States; Department of Biochemistry and Biophysics, The University of North Carolina at Chapel Hill, Chapel Hill, NC 27599, United States

## Abstract

Clustered regularly interspaced short palindromic repeats (CRISPR), widely used for gene editing, relies on bacterial endonucleases like Cas9 to study gene functions and develop therapies. However, its potential effects on mammalian cellular behavior remain unclear. Here, we systematically profiled effects of stable Cas9 expression on growth of 32 cell lines spanning 9 cancer types and non-cancerous cells, finding growth alterations in a subset. To investigate mechanisms, we established the SpCas9 interactome in DU145 and MDA-MB-231 cells, both showing Cas9-enhanced growth, and identified ribosomal proteins as the top shared interactors. RNA-seq analysis revealed that Cas9 expression in DU145 cells activated PI3K signaling. Mechanistic studies showed that ribosomal proteins, including RPL26 and RPL23a, bind to Sin1, a core mTORC2 component, leading to mTORC2 activation. Notably, SpCas9 interacts with both RPL26/RPL23a and Sin1, acting as a scaffold to stabilize their association and enhance mTORC2 activation, even in the absence of growth factors. Our study systematically characterizes Cas9’s effects on cell growth regulation and uncovers a novel Cas9-ribosome-mTORC2 signaling axis that promotes cell growth. These findings underscore the need to consider unintended cellular effects in CRISPR applications and highlight the importance of engineering safer Cas9 variants for biomedical research and clinical therapies.

## Graphical Abstract

**Graphical Abstract:**
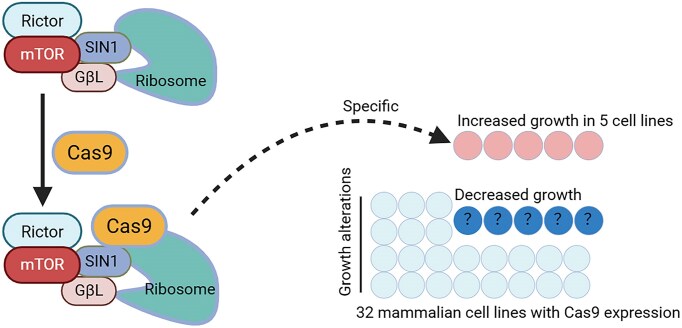

## Introduction

The CRISPR (clustered regularly interspaced short palindromic repeats) technique has revolutionized gene editing [1, 2]. Utilizing bacterial endonucleases such as *Sp*Cas9 (*Streptococcus pyogenes* Cas9) and *Sa*Cas9 (*Staphylococcus aureus* Cas9), this system enables precise and efficient genome engineering through locus-specific sgRNA(s). Cas protein-driven gene editing has become a cornerstone of biomedical research, serving as a powerful loss-of-function tool to investigate gene functions [3] and enhancer elements [4], as well as to identify potential drug targets [5, 6]. Beyond research applications, CRISPR has demonstrated its potential for correcting disease-causing mutations in mice [7–10] and dogs [11]. Notably, CRISPR-based gene editing has advanced to human clinical trials, including engineered T cells for enhanced immunotherapies [12] and treatments for β-thalassemia (NCT03655678) and sickle cell disease (NCT03745287). In a landmark achievement, the FDA has approved the first CRISPR-based therapy to correct the genetic defect underlying sickle cell disease. Excitingly, specific base and prime editing tools based on Cas9 (by fusing Cas9 with deaminases) to precisely introduce point mutations have also been developed [13] and are quickly being moved to clinical trial [14].

Because Cas9 is a prokaryotic protein absent in mammalian cells, its application in gene therapy raises safety concerns, including immune reactivity, tissue specificity, and gene editing efficiency/fidelity. Notably, human T cells recognizing bacterial Cas9 proteins have been identified [15], though the biological implications remain unclear. While constitutive or inducible Cas9 expression in mouse tissues did not cause morphological abnormalities [16], several gene-editing risks have emerged: (i) Cas9 may trigger a p53-mediated DNA damage response, enriching for cancer-prone cells [17, 18]; (ii) unexpected chromosomal deletions and genome rearrangements have been reported [19, 20]; (iii) CRISPR editing has led to thousands of LINE-1 retrotransposon insertions, suggesting unanticipated genetic modifications [21]; and (iv) genotoxic effects have been observed in base/prime editing of human hematopoietic stem cells [22]. These findings highlight the need for further evaluation and refinement of CRISPR-based gene therapies to minimize potential risks.

CRISPR-based tools, including CRISPR, CRISPRi, CRISPRa, and base editing, rely on bacterial Cas9 protein expression and activity. However, its impact on mammalian cellular responses and behavior remains unclear. Understanding these effects is essential for ensuring CRISPR’s safe application in humans and maintaining the accuracy of CRISPR-based assays, such as genetic screens, in biomedical research. Beyond Cas9, other CRISPR-associated proteins, including Cas12a [23], CasX [24], and CasY [25] from prokaryotes, as well as the eukaryotic-derived Fanzor [26], have been identified, though their potential influence on mammalian cell behavior remains untested. In this study, we systematically evaluate the effects of Cas9 expression on mammalian cell growth and uncover the molecular mechanisms underlying these changes. Our findings provide insights into mitigating unintended cellular responses, ultimately improving the safety and reliability of CRISPR technologies in both research and clinical applications.

## Materials and methods

### Cell culture and transfection

Human renal cell carcinoma cell lines 786-O, A498, RCC4, ACHN, HKC-8, UMRC2, and UMRC6, human breast cancer cell lines MDA-MB-231, MDA-MB-453, MDA-MB-468, MCF-7, and T47D; human colon cancer cell lines HCT116 and DLD1; human prostate cancer cell lines LNCaP, DU145, PC3, and 22Rv1, human pancreatic cancer cell lines AsPC-1, HuP-T3, and MIA PaCa-2, human lung cancer cell lines A549 and H1299, human Ewing sarcoma cell lines A673 and MHH-ES-1, human glioma cell lines T98G and U87MG, and human cervical cancer cell line HeLa, human immortalized kidney cell lines HEK293 and HEK293T, benign human prostate cell line BPH1, mouse embryonic fibroblasts (MEFs), and mouse embryo 10T1/2 cells were cultured in DMEM medium supplemented with 10% FBS (Fetal Bovine Serum), 100 units of penicillin, and 100 mg/ml streptomycin.

Cell transfection was carried out using Lipofectamine 3000 or polyethylenimine (PEI) as previously described [27, 28]. Lentiviral packaging for shRNA or cDNA expression and subsequent cell infection was performed following established protocols [29, 30]. After infection, cells were selected in culture media with hygromycin (200 μg/ml) or puromycin (1 μg/ml), depending on the viral vector used, for at least 72 h to eliminate non-infected cells.

### Plasmids

Cas9-Flag (plenti-CRISPR-V2) was purchased from Addgene (plasmid #52961). To generate a Cas9-negative control plasmid, the coding sequence of Cas9 was removed from the lenti-CRISPR-V2 backbone. Briefly, the Cas9 open reading frame was excised by restriction enzyme digestion using NheI and BamHI, and the resulting fragment was replaced with a short stuffer sequence containing a stop codon to maintain plasmid integrity. sgEMX-1 construct was as reported previously [29]. SuperHA-Sin1, Flag-Sin1, HA-mTOR-KD (kinase domain), superHA-Rictor, HA-Raptor, superHA-mTOR, HA-GβL, and GST-Akt1-tail constructs are as previously reported [31]. GST-RPL26 and GST-RPL23a were constructed by cloning RPL26 or RPL23a cDNA into the pCMV-GST vector using primers below:

CMV-GST-RPL23A-BamHI-F: GCATGGATCCGCGCCGAAAGCGAAG

CMV-GST-RPL23A-SalI-R: GACTGTCGACTTAGATGATCCCAATTTTGTTGGC

CMV-GST-RPL26-BamHI-F: GCATGGATCCAAGTTTAATCCCTTTGTGACTTC

CMV-GST-RPL26-SalI-R: GACTGTCGACTTATTCCTGCATCTTCTCAATGG

Sin1-N (aa 1–137), Sin1-CRIM (aa 138–266), Sin1-RBD (aa 267–376), and Sin1-PH (aa 377–522) plasmids were constructed according to a previous study [31].

sgRNAs targeting human RPL26 or RPL23a are designed as below and cloned into pLenti-CRISPR-V2 vector.

RPL26-sgRNA-1F: CACCGTGCCCTTTTCCTTTCCTACT

RPL26-sgRNA-1R: AAACAGTAGGAAAGGAAAAGGGCAC

RPL26-sgRNA-2F: CACCGAATGGCACAACTGTCCACGT

RPL26-sgRNA-2R: AAACACGTGGACAGTTGTGCCATTC

RPL26-sgRNA-3F: CACCGACCGCAAAAAGATCCTCGAA

RPL26-sgRNA-3R: AAACTTCGAGGATCTTTTTGCGGTC

RPL23a-sgRNA-1F: CACCGGAAGCTGTATGACATTGATG

RPL23a-sgRNA-1R: AAACCATCAATGTCATACAGCTTCC

RPL23a-sgRNA-2F: CACCGGCACGTCACCCACCTTCCGG

RPL23a-sgRNA-2R: AAACCCGGAAGGTGGGTGACGTGCC

RPL23a-sgRNA-3F: CACCGGAAACTTGATGATAGCATAG

RPL23a-sgRNA-3R: AAACCTATGCTATCATCAAGTTTCC

### Immunoblot and immunoprecipitation analyses

Cells were lysed in EBC buffer (50 mM Tris, pH 7.5, 120 mM NaCl, 0.5% NP-40) or RIPA buffer (50 mM Tris, pH 7.5, 150 mM NaCl, 1% Triton X-100, 1% sodium deoxycholate, 0.1% SDS), both supplemented with protease inhibitor (PI) cocktails and phosphatase inhibitor cocktails. The protein concentrations of the whole cell lysates were determined using the Bio-Rad Bradford protein assay and measured with a NanoDrop OneC. Equal amounts of whole cell lysates were resolved by sodium dodecyl sulfate–polyacrylamide gel electrophoresis (SDS-PAGE) and subjected to immunoblotting with the indicated antibodies.

For immunoprecipitation (IP) analyses, unless otherwise specified, 1 mg of lysate was incubated with agarose beads coupled to antibodies specific for the tag for 3-4 h at 4°C. For endogenous IPs, the incubation of cell lysates with antibodies was extended overnight, followed by another 1 h with Protein A/G XPure agarose resins. The immuno-complexes were washed four times with NETN buffer [20 mM Tris, pH 8.0, 100 mM NaCl, 1 mM ethylenediamine tetraacetic acid (EDTA), 0.5% NP-40] before being resolved by SDS–PAGE and analyzed by immunoblotting with the indicated antibodies.

Notably, all uncropped, unprocessed western blotting images are included in Supplementary Fig. S5.

### T7E1 gene editing assays

EMX-1 sgRNA was transfected into the indicated DU145 or MDA-MB-231 cells. Three days post-transfection with the indicated DNA constructs, cells were harvested, and genomic DNA was extracted using QuickExtract™ DNA Extraction Solution (Biosearch Technology SS000035-D2) according to manufacturer’s instructions. End-point polymerase chain reaction (PCR) was performed using 10 μl of genomic DNA as the template, with the indicated primers and high-fidelity Taq DNA polymerase (M7122, Promega). PCR products were verified by DNA electrophoresis in 1× TAE buffer and purified using PCR cleanup kits (BS664, Bio Basic, Inc.). PCR products from control or Cas9-expressing cells (600–800 ng) were mixed, denatured, and annealed to generate small indels in the hybridized products. One microliter of T7 Endonuclease I (NEB M0689) was added, and the reactions were incubated at 37°C for 1 h. Digested PCR products were resolved by 2% TAE agarose gel electrophoresis. Indel frequencies were calculated by comparing the intensities of the T7-digested bands to the sum of both full-length and digested bands, quantified using ImageJ.

### mRNA translation assays

Messenger RNA (mRNA) translation assays were performed using the Invitrogen Click-iT™ HPG Alexa Fluor™ 488 Protein Synthesis Assay Kit (cat# C10428) according to manufacturer’s instructions. Briefly, sub-confluent cells were plated in six-well plates overnight. Where indicated, 200 μg/ml CHX was used to treat cells overnight as a negative control (NC). To prepare the Click-iT^®^ HPG (Component A), it was diluted 1:1000 in L-methionine-free medium to a final working concentration of 50 μM. The media was removed from the cells, and 100 μl/well of medium containing the 50 μM Click-iT^®^ HPG working solution was added to each well for 30 min. Cells were then washed with sterile 1× PBS, and 100 μl/well of 3.7% formaldehyde in PBS was added for fixation. The cells were incubated at room temperature for 15 min, after which the fixative was removed, and the cells were washed twice with 3% bovine serum albumin (BSA) in PBS. Next, 100 μl/well of 0.5% Triton X-100 in PBS was added, and cells were incubated at room temperature for 20 min. Following two additional washes with 100 μl/well of 3% BSA in PBS, 100 μl/well of Click-iT^®^ reaction cocktail was added and incubated for 30 min at room temperature in the dark. For nuclear staining, the HCS NuclearMask™ Blue Stain (Component G) solution was diluted 1:2000 in PBS to obtain a 1× working solution, which was added at 100 μl/well. Cells were incubated for 30 min at room temperature, protected from light. After two washes, imaging was performed, and image intensity analysis was conducted.

### Colony formation assays

Indicated cells were seeded into six-well or 6 cm dishes (500 or 1,000 cells per well) and cultured in a 37°C incubator with 5% CO_2_ for 7–21 days until visible colonies formed. Colonies were gently washed with PBS, fixed with methanol for 30 min, and stained with 0.5% crystal violet for 30 min. After staining, colonies were washed with distilled water and air-dried. Colony numbers were counted manually. Three independent biological experiments were performed to generate error bars.

### Cell viability MTT assays

Cell viability MTT assays were performed as described previously [32]. Briefly, 3,000 indicated cells were seeded into each well of a 96-well plate for MTT assays to assess cell viability at the indicated time points, following a method adapted from Thermo Fisher MTT Assay Protocol. Briefly, at the indicated time points post-seeding, 10 μl of MTT solution was added to each well and incubated in a culture incubator (37°C with 5% CO_2_) for 4 h. The medium was then removed, and 100 μl of DMSO (DiMethyl SulfOxide) was added to each well to dissolve the formazan crystals, followed by incubation for 10 min at 37°C. After thorough mixing, absorbance at 540 nm was measured using the BioTek Cytation 5 Cell Imaging Reader.

### Mass spectrometry analyses to identify flag-SpCas9 interacting proteins

NC and SpCas9-Flag lentiviruses were used to infect DU145 and MDA-MB-231 cells, respectively. Infected cells were selected in media containing 1 μg/ml puromycin for 72 h to eliminate non-infected cells. The resulting cells were harvested using EBC buffer (50 mM Tris, pH 7.5, 120 mM NaCl, 0.5% NP-40) supplemented with a PI and protein phosphatase inhibitor (PPI) cocktail. For IP, 2 mg of whole-cell lysate (WCL) was incubated with Flag-M2 agarose beads under gentle rotation at 4°C for 4 h. The Flag immunoprecipitants were washed four times with NETN buffer (20 mM Tris, pH 8.0, 100 mM NaCl, 1 mM EDTA, and 0.5% NP-40). For MDA-MB-231 cells, proteins were diluted to 100 μg/μl using 8 M urea (U4883, Sigma–Aldrich) and subjected to FASP trypsin digestion. Briefly, proteins were reduced with 50 mM DTT (A39255, Thermo Fisher Scientific) for 15 min at 65°C, followed by dilution with 200 μl of 8 M urea. The samples were transferred to a 30K MWCO spin filter (14‐558‐349, Thermo Fisher Scientific) and centrifuged at 10 000 × *g* for 30 min at room temperature. Proteins were washed twice with 200 μl of 8 M urea under the same conditions. Alkylation was performed using 100 μl of 15 mM 2‐chloroacetamide (148415000, Thermo Fisher Scientific) in 8 M urea for 20 min in the dark at room temperature. The spin filter was washed twice at 10 000 × *g* for 20 min, followed by buffer exchange with 50 mM ammonium bicarbonate (ABC), pH 8.0, by centrifugation at 10 000 × *g* for 15 min. Trypsin digestion was carried out by adding 100 μl of 50 mM ABC and 2.5 μg of trypsin (V511C, Promega) to the spin filter, followed by incubation at 37°C for 18 h. Peptides were recovered in a new receiver tube by centrifugation at 10 000 × *g* for 15 min and eluted twice using 50 μl of 0.5% TFA in water at 10 000 × *g* for 10 min. The samples were concentrated to 100 μl using a Savant™ SPD131DDA SpeedVac Concentrator (Thermo Fisher Scientific), followed by C18 column desalting (89870, Thermo Fisher Scientific). The samples were then concentrated and resolubilized in 100 μl of LC‐Optima MS‐grade water using the SpeedVac. Ethyl acetate extraction was performed to remove residual detergents, followed by another round of SpeedVac concentration. Peptide quantification was performed using the QFP (quantitative peptide assays and standards) kit (23290, Thermo Fisher Scientific). Detailed liquid chromatography-mass spectrometry/mass spectrometry (LC-MS/MS) methods and data filtering approaches were described previously [29]. For DU145 cells, beads were washed 3× with 1× PBS and then reconstituted in 2 M urea (U4883, Sigma–Aldrich), 50 mM Tris pH 8. Proteins were reduced with 5 mM DTT for 30 min at 37°C and then alkylated with 50 mM CAA for 20 min at RT in the dark. The proteins were then digested overnight with trypsin at 37°C. The samples were dried followed by C18 column desalting (ZTC18S096, Sigma). Dried-down peptides were reconstituted for LC-MS analysis.

For MDA-MB-231 cells, peptides were separated using reverse-phase nano-HPLC using a nanoACQUITY ultraperformance LC (Waters Corporation). Peptides were trapped on a 2 cm column (Pepmap 100; 3-μm particle size and 100-Å pore size) and separated on a 25 cm EASYspray analytical column (75-μm inside diameter [i.d.], 2.0-μm C_18_ particle size, and 100-Å pore size) at 300 nl/min and 35°C, respectively. For peptide separation and elution, mobile phase A was 0.1% formic acid (FA) in water, and mobile phase B (MPB) was 0.1% FA in acetonitrile. A one-step 180-min gradient of 2% to 25% MPB was followed by an increase to 90% B over 3 min and column washing and equilibration. MS analysis was performed on an Orbitrap Fusion Lumos mass spectrometer (Thermo Scientific) operated in data-dependent acquisition mode. The MS1 scans were acquired in the Orbitrap at 120K resolution with a 1 × 10^6 automated gain control (AGC) target, 100 ms max injection time, and a 350–2000 m/z scan range. MS2 targets were filtered for charge states 2–7, with a dynamic exclusion of 30 s, and were accumulated using a 1.6 m/z quadrupole isolation window. For charge state 2, MS2 scans were performed in the ion trap with rapid scan rate and CID fragmentation at 30% NCE, AGC target 4e3, and a max injection time of 250 ms. For charge states 3–7 with a minimum intensity of 500 000, MS2 scans were performed in the Orbitrap at 7500 resolution with HCD fragmentation at 30% NCE, AGC target 5e4, and a max injection time of 22 ms. For charge states 3–7 with a maximum intensity <500 000, MS2 scans were performed in the ion trap with rapid scan rate and CID fragmentation at 30% NCE, AGC target 4e3, and a max injection time of 250 ms. The cycle time was set at 3s. For DU145 cells, peptides were separated using reverse-phase nano-HPLC using an Ultimate 3000 RSLCnano system (Thermo Scientific). Peptides were trapped on a μPAC™ Trapping Column (Thermo Scientific) and separated on a 50 cm μPAC™ Neo HPLC Column (Thermo Scientific). Peptides were injected onto the trap column at 10 μl/min for 3 min using the loading pump. Initially the nanoflow rate was set at 0.75 μl/min and 2% MPB while the peptides were loaded onto the trap column; at 2.8 min the solvent composition was changed to 10% MPB. At 5 min the flow rate was dropped to 0.300 μl/min at 12% MPB. A two-step gradient was used from 12% to 20% MPB for 41.8 min followed by 20% to 40% MPB for 15.9 min. The flow rate was then increased to 0.750 μl/min for column washing using seesaw gradients and re-equilibration. MS analysis was performed on an Orbitrap Eclipse Tribrid mass spectrometer (Thermo Scientific) operated in data-dependent acquisition mode. The MS1 scans were acquired in the Orbitrap at 120K resolution with a 1 × 10^6 AGC target, auto max injection time, and a 375–2000 m/z scan range. MS2 targets were filtered for charge states 2–7, with a dynamic exclusion of 60 s, and were accumulated using a 0.7 m/z quadrupole isolation window. MS2 scans were performed in the ion trap at a rapid scan rate following higher energy collision dissociation with a 35% normalized collision energy. MS2 scans used a 1 × 10^4 AGC target and 70 ms max injection time. The cycle time was set at 3 s. MDA-MB-231 data were searched with MaxQuant (version 1.6.6.7) [33, 34] with a FASTA-reviewed Homo Sapiens database. Data analyses were performed in Perseus (version 1.6.3.4). DU145 data were searched in MaxQuant (2.6.7.0) [33, 34] with a FASTA-reviewed Homo Sapiens database containing 20 436 sequences downloaded on 10 June 2024 and appended with custom construct sequences. Data analyses were performed with custom scripts in R. The mass spectrometry proteomics data have been deposited to the ProteomeXchange Consortium via the PRIDE [35] partner repository with the dataset identifier PXD064062.

### RNA-seq analysis

DU145 cells stably expressing NC or WT-SpCas9 via lentiviral infection were harvested at sub-confluent confluence. Bulk RNA was extracted using EZ-10 DNAaway RNA Miniprep Kit (Biobasic BS88136) and subjected to RNA library construction and subsequent high-throughput sequencing by Novogene, Inc. Bioinformatics analyses were also performed by Novogene. A brief bioinformatics analysis pipeline from Novogene is listed below.

- Data quality control: Raw data (raw reads) of fastq format were firstly processed through fastp software. In this step, clean data (clean reads) were obtained by removing reads containing adapters, reads containing poly-N, and low-quality reads from raw data. At the same time, Q20, Q30, and GC content of the clean data were calculated. All the downstream analyses were based on the clean data with high quality.
- Reads mapping to the reference genome: Reference genome and gene model annotation files were downloaded from genome website. Use HISAT2 (2.2.1) to build the index of the reference genome and use HISAT2 to align paired-end clean reads to the reference genome. HISAT2 can use the gene model annotation file to create splice-aware alignments, providing better alignment accuracy compared to other non-splice alignment tools.
- Quantification of gene expression level feature: Counts (2.0.6) was used to count the number of reads mapped to each gene. And then FPKM of each gene was calculated based on the length of the gene and reads count mapped to this gene. FPKM, expected number of Fragments Per Kilobase of transcript sequence per Million base pairs sequenced, considers the effect of sequencing depth and gene length for the reads count at the same time, and is currently the most used method for estimating gene expression levels.
- Differential expression analysis: For DESeq2 with biological replicates: Differential expression analysis for two conditions/groups was performed using the DESeq2 R package (1.42.0). DESeq2 provides statistical programs for determining differential expression in digital gene expression data using models based on negative binomial distribution. The resulting *P*-value is adjusted using the Benjamini–Hochberg methods to control the error discovery rate. The threshold of significant differential expression: padj ≤ .05 & |log_2_[fold change (FC)]| ≥ 1. For edgeR without biological replicates: Prior to differential gene expression analysis, for each sequencing library, read counts were adjusted using the edgeR R package (4.0.16) by scaling normalization factors to eliminate differences in sequencing depth between samples, followed by differential expression analysis. The resulting *P*-value is adjusted using the Benjamini–Hochberg methods to control the error discovery rate. The threshold of significant differential expression: padj ≤ .05 & |log_2_(FC)| ≥ 1.
- GO and KEGG enrichment analysis of differentially expressed genes: Gene Ontology (GO) enrichment analysis of differentially expressed genes was implemented by the clusterProfiler (4.8.1), in which gene length bias was corrected. GO terms with corrected *P*-value <.05 were considered significantly enriched by differentially expressed genes. KEGG (Kyoto Encyclopedia of Genes and Genomes) is a database resource for understanding high-level functions and utilities of the biological system, such as the cell, the organism, and the ecosystem, from molecular-level information, especially large-scale molecular datasets generated by genome sequencing and other high-throughput experimental technologies (http://www.genome.jp/kegg/). We used clusterProfiler R package to test the statistical enrichment of differential expression genes in KEGG pathways.
- Gene set enrichment analysis: Gene set enrichment analysis (GSEA) is a computational approach to determine whether a predefined gene set can show a significant consistent difference between two biological states. The genes were ranked according to the degree of differential expression in the two samples, and then the predefined gene set was tested to see whether they were enriched at the top or bottom of the list. GESA can include subtle expression changes. We used the local version of the GSEA analysis tool, http://www.broadinstitute.org/gsea/index.jsp, GO and KEGG gene sets were used for GSEA independently.

### Protein purification

GST-RPL23a and GST-Akt1-tail were expressed in *Escherichia coli* BL21 (DE3) cells grown in LB medium supplemented with 150 μg/ml ampicillin at 37°C until reaching an OD_600_ of 0.5. Protein expression was induced with 0.3 mM IPTG (Isopropyl β-D-1-thiogalactopyranoside) at 16°C for 18 h. Cells were resuspended in lysis buffer containing 150 mM Tris–HCl (pH 7.5), 150 mM NaCl, 5 mM β-mercaptoethanol (BME), and 1 mM PMSF. Lysis was performed using an ultrasonic processor, and the lysate was clarified by centrifugation at 10 000 × *g* for 30 min. The supernatant was incubated with glutathione agarose beads for 1 h at 4°C, followed by three washes with ice-cold sterile 1× PBS. GST-tagged proteins were eluted using a buffer containing 50 mM Tris–HCl (pH 8.0), 10 mM glutathione, and 150 mM NaCl.

### *In vitro* mTORC2 kinase assays

*In vitro* mTORC2 kinase assays were performed as previously described [31]. Briefly, HA-IPs in CHAPS buffer was conducted using HEK293 cells transfected with superHA-Sin1 under either serum-starvation (12 h) or normal culture conditions. HA-Sin1 immunoprecipitants (IPs) were extensively washed with CHAPS buffer and used as the kinase source for the *in vitro* kinase assays. For the kinase reactions, 10 μl of HA-Sin1 IPs were incubated with 2 μg of GST-AKT1-tail (amino acids 409–480) and 200 μM cold ATP in kinase assay buffer (50 mM HEPES, pH 7.5, 10 mM MnCl_2_, 10 mM MgCl_2_, 2 mM DTT, 0.5 mM EGTA) at 37°C for 1 h. The reactions were gently mixed every 10 min and terminated by adding SDS-containing lysis buffer. Samples were resolved by SDS–PAGE, and phosphorylation of GST-AKT1-tail was detected by Western blotting using the Akt-pS473 antibody.

### *In vitro* protein binding assays

For *in vitro* binding assays, HA-Sin1 IPs in EBC buffer were collected using HEK293 cells transfected with superHA-Sin1 DNA construct under normal cell culture conditions and extensively washed with EBC buffer for four times. Resulted HA-Sin1-IPs on agarose beads were used as the bait for the binding assays. Ten microliters of HA-Sin1 IPs were incubated with 10 μg of GST-RPL23a proteins with/without 1 μg of recombinant Cas9 proteins in EBC buffer at 4°C for overnight. IPs were washed with EBC buffers before being resolved by SDS–PAGE and immunoblotted with indicated antibodies.

### Statistical analysis

Statistical analyses were performed using GraphPad Prism 8 software. A *P*-value of ≤ .05 was considered statistically significant. Data are presented as mean ± SD from at least two or three independent experiments, as indicated in the figure legends. Differences between control and experimental groups were assessed using a *t*-test, one-way ANOVA, or two-way ANOVA, as appropriate.

*Key resources table*

**Table:** 

REAGENT or RESOURCE	SOURCE	IDENTIFIER
Beads and Recombinant Proteins		
Glutathione agarose beads	GE Healthcare	cat#17-0756-05
Nickel HTC agarose beads	GoldBio	cat#R-202-100
Anti-HA agarose beads	Millipore Sigma	cat#A-2095
Anti-Flag agarose beads	Millipore Sigma	cat#A2220
Recombinant Cas9 protein	Millipore Sigma	cat#CAS9PROT
Chemicals/Compounds		
cycloheximide	Selleck	cat#S6611
MG132	Selleck	cat#S2619
Protease inhibitor cocktail	Apexbio Technology	cat#K1008
Phosphatase inhibitor cocktail	Apexbio Technology	cat#K1015
Torin 2	MilliporeSigma	cat#SML1244
MK2206	MilliporeSigma	cat#12405
Rapamycin	MilliporeSigma	cat#553210
S6K-I	MilliporeSigma	cat#PZ0143
Antibodies		
Anti-HA antibody	Cell Signaling Technology	cat#3724
Anti-NRF2 antibody	abcam	cat#ab62352
Anti-KEAP1-antibody	Cell Signaling Technology	cat#8047
Anti-Akt-pS473 antibody	Cell Signaling Technology	cat#4060
Anti-Akt-pT308 antibody	Cell Signaling Technology	cat#9275
Anti-S6K-pT389 antibody	Cell Signaling Technology	cat#9205
Anti-mTOR antibody	Cell Signaling Technology	cat#2972
Anti-Rictor antibody	Cell Signaling Technology	cat#2114
Anti-Sin1 antibody	Cell Signaling Technology	cat#12860
Anti-Akt1 antibody	Cell Signaling Technology	cat#2938
Anti-Akt (pan) antibody	Cell Signaling Technology	cat#4691
Anti-Cas9 antibody	Cell Signaling Technology	cat#19526
Anti-RPL26 antibody	Cell Signaling Technology	cat#5400
Anti-GSK3β-pS9	Cell Signaling Technology	cat#5558
Anti-FOXO1-pT24/FOXO3a-pT32	Cell Signaling Technology	cat#2599
Anti-RPL23a antibody	Proteintech	cat#16386–1-AP
Anti-GAPDH antibody	Santa Cruz Biotechnology	cat#sc-47 724
Anti-GST antibody	Santa Cruz Biotechnology	cat#sc-459
Anti-Vinculin antibody	Santa Cruz Biotechnology	cat#sc-25336
Anti-Flag antibody	Millipore Sigma	cat#F1804
Anti-Flag antibody	Millipore Sigma	cat#F7425
Anti-Tubulin antibody	Millipore Sigma	cat#T5168
Anti-HA antibody	Proteintech	cat#51064-2-AP
Anti-His-Tag antibody	Proteintech	cat#66005-1-Ig
Transfection Reagents and Antibiotics		
Lipofectamine 3000	Thermo Fisher Scientific	cat# L3000150
Polyethylenimine (PEI)	Polysciences, Inc.	cat# 23866-1
Puromycin	Fisher BioReagents	cat# 58-58-2
Hygromycin	Sigma–Aldrich	cat# H3274
Experimental Models: Cell Lines		
786-O	Dr Qing Zhang (UT Southwestern)	
A498	ATCC	Cat# HTB-44
ACHN	ATCC	Cat#CRL-1611
RCC4	Dr Williams Kim (UNC)	
UMRC2	Dr Williams Kim (UNC)	
UMRC6	Dr Williams Kim (UNC)	
HKC-8	Dr Williams Kim (UNC)	
MDA-MB-231	ATCC	Cat# CRM-HTB-26
MDA-MB-453	Dr Qing Zhang (UT Southwestern)	
MDA-MB-468	Dr Qing Zhang (UT Southwestern)	
MCF-7	ATCC	
T47D	Dr Qing Zhang	
HCT116	Dr Gabrielle Rupprecht (Duke University)	
DLD1	Dr Gabrielle Rupprecht (Duke University)	
LNCaP	Dr Greg Wang (Duke University)	
DU145	Dr Greg Wang (Duke University)	
PC3	Dr Haojie Huang (Mayo Clinic)	
22Rv1	Dr Haojie Huang (Mayo Clinic)	
BPH1	Dr Haojie Huang (Mayo Clinic)	
AsPC-1	Dr Jenjen Yeh (UNC)	
HuP-T3	Dr Jenjen Yeh (UNC)	
MIA PaCa-2	Dr Jenjen Yeh (UNC)	
A549	Dr Chad Pecot (UNC)	
H1299	Dr Chad Pecot (UNC)	
A673	Dr Ian J Davis (UNC)	
MHH-ES-1	Dr Ian J Davis (UNC)	
T98G	Dr Ryan Miller (UNC)	
U87MG	Dr Ryan Miller (UNC)	
HeLa	Dr Wenyi Wei (BIDMC)	
MEF	Dr Wenyi Wei (BIDMC)	
HEK293	ATCC	Cat# CRL-1573
HEK293T	Dr Wenyi Wei (BIDMC)	
10T1/2	Dr Mack, Christopher (UNC)	
Software		
FlowJo 10.8.1	Tree Star	
Graphpad Prism 8	Prism	
Others		
QuickExtract DNA Extraction Solution	Bioresearch technologies	cat#QE09050
DNA Clean & Concentrator-5	Zymo Research	cat#D4013
Matrigel	Corning	cat#356234
Deposited Data		
Original microscope images and uncropped western blot images can be found at the DOI URL:		

## Results

### Profile the effects of stable SpCas9 expression on the growth of 32 cell lines across 9 cancer types and 4 non-cancerous lines

**Figure 1.**
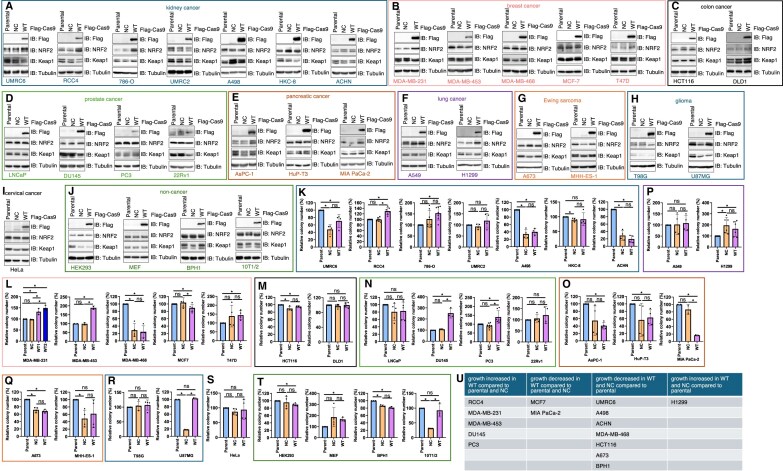
Profiling effects of stable SpCas9 expression on cell growth. (**A**–**J**) Immunoblot (IB) analysis of WCL from indicated cells. Where indicated, Parental represents parental cells, NC stands for negative control expressing NC, and WT represents WT-Cas9-Flag expression. Indicated cells were infected with indicated lentiviruses for 24 h and selected with 1 μg/ml puromycin for at least 72 h to eliminate non-infected cells prior to cell harvest and analysis. (**K**–**T**) Quantifications of 2D colony formation results. Error bars were calculated as mean ± SD; (*n*) biological replicates are as indicated in each panel. *P< .05 (one-way ANOVA test), ns: not significant. (**U**) A table summarizing cell lines whose cell growth is affected by stable SpCas9 expression.

To investigate whether Cas9 expression modulates mammalian cell behavior, we established stable Cas9-expression in 32 mammalian cell lines using lentiviral infection following standard protocols [36]. This process took much longer than anticipated due to unexpected viral infection effects, as some cell lines were particularly sensitive to. To address this, we included an NC using the same viral vector backbone containing the gRNA scaffold but only lacking the Cas9 coding sequence. As a result, we generated three isogenic lines for each of the 32 cell lines: parental, NC, and Cas9-expressing cells, with Flag-Cas9 expression confirmed in each (Fig. 1A–J). These 32 cell lines represent 10 major cancer types, including kidney (Fig. 1A), breast (Fig. 1B), colon (Fig. 1C), prostate (Fig. 1D), pancreatic (Fig. 1E), lung (Fig. 1F), Ewing sarcoma (Fig. 1G), glioma (Fig. 1H), cervical cancer (Fig. 1I), and non-cancerous cells (Fig. 1J). Previously, we reported that SpCas9 serves as a substrate for the endogenous E3 ubiquitin ligase Keap1, potentially stabilizing Keap1 substrates through competitive binding [29]. Consistent with this, SpCas9 expression led to the stabilization of the known Keap1 substrate NRF2 in multiple cell lines, including UMRC6, RCC4, and 786-O (Fig. 1A); MDA-MB-231 and T47D (Fig. 1B); AsPC-1 and MIA-PaCa-2 (Fig. 1E); and H1299 (Fig. 1F).

We then performed 2D colony formation assays to assess the effects of stable Cas9 expression on cell growth *in vitro* with multiple biological replicates by at least two independent researchers in lab (Fig. 1K–T and Supplementary Fig. S1). Out of 32 cell lines tested, NC and SpCas9 expression marginally affected growth of 7 cell lines, including UMRC2, A549, DLD1, LNCaP, 22RV1, T98G, and HeLa. In addition, NC viral infection alone significantly reduced the growth of 11 cell lines, including UMRC6, A498, HKC-8, ACHN, MDA-MB-468, HCT116, A673, MHH-ES-1, U87MG, BPH1, and 10T1/2. Additionally, in cell lines where NC infection did not impact growth, SpCas9 expression led to increased growth in five cell lines (RCC4, MDA-MB-231, MDA-MB-453, DU145, and PC3), but decreased growth in two lines (MCF-7 and MIA PaCa-2) (Fig. 1U). For AsPC-1, HuP-T3, and HEK293 cells, the effects of NC infection were complicating the interpretations, although Cas9 expression reduced growth compared with their respective parental cells. Notably, these growth changes did not always correlate with NRF2 expression (Fig. 1), suggesting that NRF2 is unlikely to be the only underlying mechanism.

### SpCas9 interactome analysis reveals ribosomal proteins as major binding partners in DU145 and MDA-MB-231 cells

**Figure 2.**
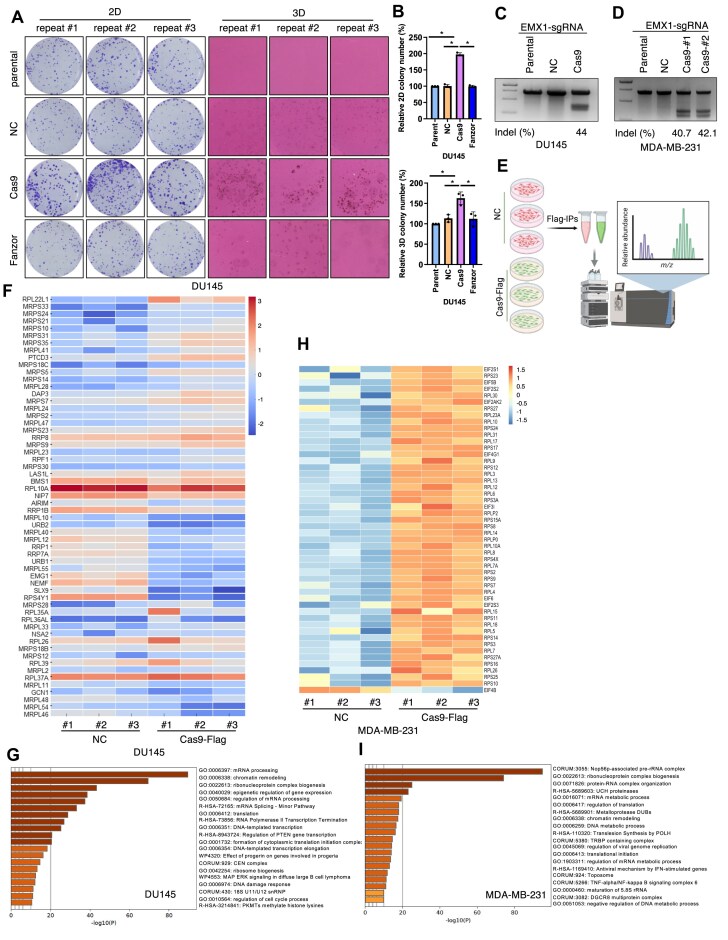
SpCas9 interactome identifies ribosome proteins as top shared hits. (**A**) Representative 2D colony formation and 3D soft agar results from indicated DU145 cells and quantified in (**B**). Error bars were calculated as mean ± SD; (n) biological replicates are as indicated in each panel. *P < .05 (one-way ANOVA test). (**C**, **D**) Representative T7E1 assay results using sgEMX-1 in indicated cells. (**E**) A cartoon illustration of the procedure for Flag-IPs in indicated cells followed by sample processing and mass spectrometry analysis to define SpCas9 binding proteins. A heatmap showing top SpCas9 binding proteins in DU145 cells (**F**) or MDA-MB-231 cells (**H**). (**G**, **I**) GO term analysis results from panels (F) and (H) showing major biological processes.

To find reasons for Cas9 expression-induced cell growth changes beyond Keap1 suppression as we reported previously [29], we focused on two cell lines that showed increased cell growth upon Cas9 expression, including DU145 (Fig. 1N) and MDA-MB-231 (Fig. 1L). We have confirmed that stable expression of Cas9 but not NC increased growth of both DU145 cells *in vitro* (Fig. 2A and B). In addition, we also confirmed that stable Cas9 expression in either DU145 (Fig. 2C) or MDA-MB-231 (Fig. 2D) cells led to efficient gene editing ability, assuring the level of Cas9 expression is suitable for gene editing. Notably, expressing the eukaryotic gene-editing endonuclease Fanzor (Supplementary Fig. S2A) failed to affect DU145 cell growth (Fig. 2A). We then performed Flag-Cas9-IPs followed by mass spectrometry analyses in DU145 or MDA-MB-231 cells stably expressing either NC or Flag-Cas9 (Fig. 2E and Supplementary Fig. S2B). Using a two-fold increase |log_2_(FC)| ≥ 1 and a *P*-value <.05 [−log_10_(*P*-value) >1.3] as a cutoff, we observed 500 and 200 significantly enriched Cas9-specific binding proteins in DU145 and MDA-MB-231 cells, respectively, after removing common contaminating proteins (Supplementary Fig. S2C and D). These proteins belong to distinct biological pathways ranging from cellular matrix to metabolic signaling with the overlapped top hits from two cell lines being ribosome components, including both 60S (RPL) and 40S (RPS) proteins (Fig. 2F–I). We hypothesized that depending on cellular context, Cas9 may interact with distinct ribosome proteins to regulate cell growth. Notably, a recent study also identified ribosomal proteins as major Cas9-interacting partners in postmitotic neurons [37].

### SpCas9 binds ribosomal proteins to facilitate mTORC2/Akt signaling activation in promoting cell growth

**Figure 3.**
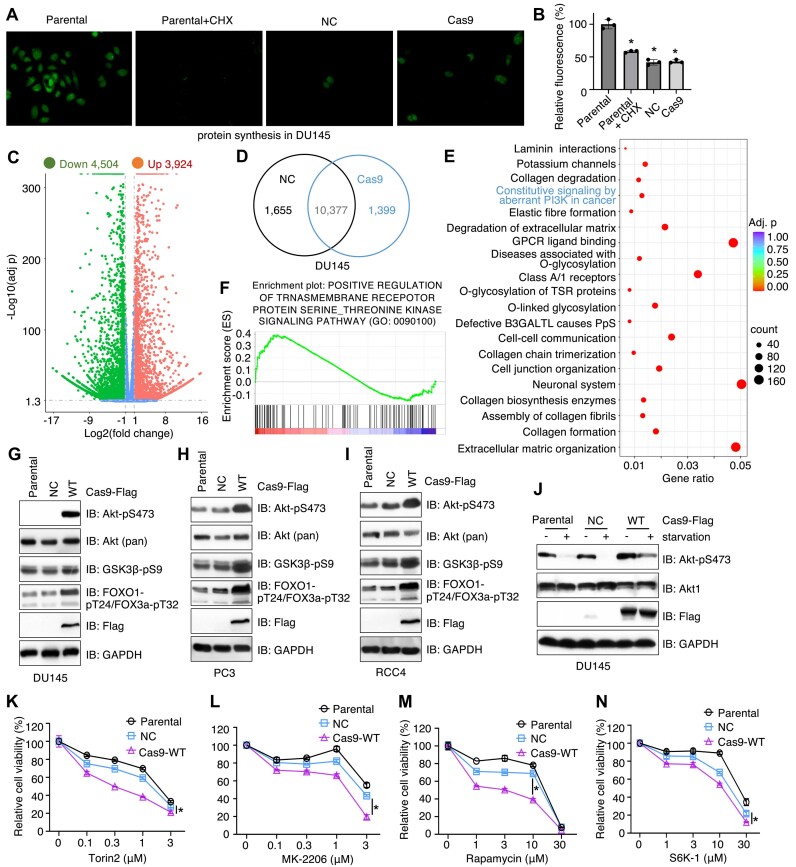
SpCas9 binds ribosomes to regulate mTORC2 activation with minimal effects on mRNA translation efficiency. (**A**, **B**) Representative confocal microscopy images showing mRNA translation in indicated cells. Where indicated, 200 μg/ml CHX is used to treat cells for 6 h, and the GFP signal intensities are quantified in panel (B). Error bars were calculated as mean ± SD, *n* = 3 biological replicates. *P< .05 (one-way ANOVA test). (**C**) A volcano plot showing significantly upregulated (red) or downregulated (green) gene expression in Cas9-expressing DU145 cells compared with NC-expressing DU145 cells. -log_10_(adj *P*) value ≤1.3 as a cutoff. (**D**) A Venn diagram showing the number of genes with significant expression changes in indicated DU145 cells. (**E**) Pathway analyses as indicated on cellular processes on genes altered in Cas9 versus NC DU145 cells. Gene counts are represented by sizes of dots and statistical significance is indicated by warm/cold color as indicated. (**F**) A GESA analysis on Cas9 versus NC altered gene expression indicating an enrichment in protein serine/threonine kinase signaling. (**G**–**J**) IB analysis of WCL from indicated cells. Where indicated in panel (J), cells were serum-starved for 12 h before cell collection. (**K**–**N**) Representative cell viability assays of indicated cells treated with indicated compounds for 72 h. Error bars were calculated as mean ± SD, *n* = 3 biological replicates. *P< .05 (one-way ANOVA test).

To further investigate how Cas9 facilitates cell proliferation through novel binding partners, we focused on ribosomal proteins, which emerged as top interactors. To assess whether Cas9 influences ribosome-associated biological functions, we first examined mRNA translation. Using a fluorescence-based assay, we found that NC infection alone significantly reduced mRNA translation in DU145 cells, while Cas9 expression did not further diminish translation intensity (Fig. 3A and B). Despite Cas9 interacting with numerous ribosomal proteins (Fig. 2E), the fact that Cas9, but not NC, enhanced DU145 cell growth (Fig. 1N) suggests that Cas9 is unlikely to regulate cell growth by modulating ribosome-mediated mRNA translation.

To investigate how Cas9 binding to ribosomes influences cell growth, we performed RNA-seq analysis in DU145 cells expressing Cas9 or NC. Using an adjusted *P*-value < .05 and |log_2_(FC)| ≥ 1 as cutoffs, we identified 3,924 upregulated and 4,504 downregulated genes in Cas9-expressing DU145 cells (Fig. 3C). Among these, 1,399 genes were uniquely expressed in the Cas9-expressing cells (Fig. 3D). GO (Supplementary Fig. S3A) and KEGG pathway (Supplementary Fig. S3B) analyses of Cas9-induced transcriptomic changes revealed alterations in distinct cellular functions, including membrane-associated channel activities and focal adhesion. Notably, “signaling receptor activator activity” (Supplementary Fig. S3A) and the “PI3K-Akt signaling pathway” (Supplementary Fig. S3B) were highlighted in both analyses. These pathways also appeared among the top hits in Reactome analysis, including “constitutive signaling by aberrant PI3K in cancer” (Fig. 3E). GSEA further revealed significant enrichment of the pathway “positive regulation of transmembrane receptor protein serine/threonine kinase signaling” (Fig. 3F). Together, these transcriptomic data suggest that Cas9 expression may be associated with enhanced PI3K/mTORC2/Akt signaling activity.

This prompted us to investigate additional ribosomal functions in cell growth regulation, particularly the role of ribosomal association in activating mTORC2 [38]. The mTORC2/Akt signaling pathway is well-documented to promote cell growth as a PI3K downstream signaling [39]. Notably, previous studies have shown that serum starvation (via growth factor depletion) disrupts ribosomal protein binding to mTORC2, whereas growth factor stimulation restores this association and activates mTORC2 [38]. To determine whether Cas9 modulates mTORC2 signaling, we selected three cell lines, including DU145, PC3, and RCC4, where Cas9 expression consistently enhanced cell growth (Fig. 1). The growth-promoting effect of Cas9 in PC3 cells was independently validated (Supplementary Fig. S3C), and its genome editing activity was confirmed (Supplementary Fig. S3D and E). We then examined Akt phosphorylation at Ser473 (Akt-pS473), a well-characterized mTORC2 substrate, in parental, NC, and Cas9-expressing cells. In all three cell lines, expression of Cas9, but not NC, increased Akt-pS473 levels and Akt substrate phosphorylation (Fig. 3G and I and Supplementary Fig. S3F). Importantly, serum starvation markedly reduced Akt-pS473 levels in parental and NC-expressing DU145 cells, consistent with mTORC2 inactivation. However, Cas9 expression robustly maintained mTORC2/Akt-pS473 signaling even under starvation conditions (Fig. 3J), and a similar effect was observed in PC3 cells (Supplementary Fig. S3G). This unexpected yet reproducible finding suggests that Cas9 may act as a “glue” stabilizing the interaction between ribosomal proteins and mTORC2 even under serum-starved conditions when ribosomal binding to mTORC2 would typically be disrupted. This sustained activation of Akt could underlie Cas9’s role in promoting cell growth. Additionally, Cas9-expressing DU145 cells, with elevated mTORC2/Akt activation, exhibited increased sensitivity to the inhibition of mTORC2/Akt signaling by the mTOR inhibitor Torin 2 (Fig. 3K), the Akt inhibitor MK2206 (Fig. 3L), the mTORC1 inhibitor rapamycin (Fig. 3M), and the S6K inhibitor S6K-I (Fig. 3N). These data suggest Cas9-enhanced cell growth may largely depend on mTORC2/Akt signaling.

### SpCas9 binds Sin1 in mTORC2 to bridge ribosome association for mTORC2 activation

**Figure 4.**
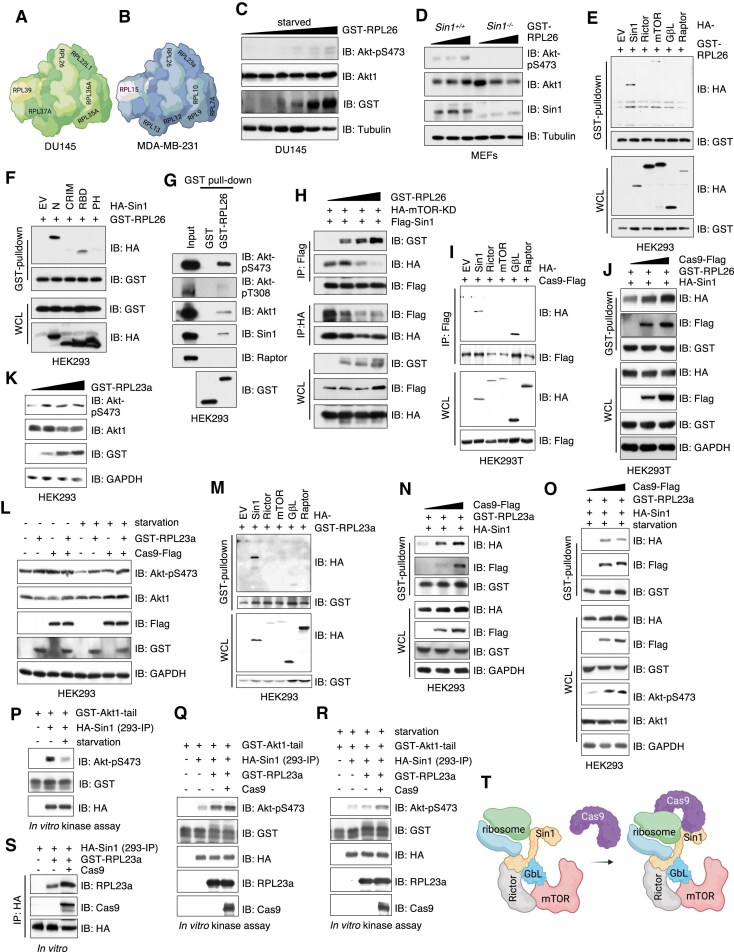
SpCas9 enhances ribosome association with mTORC2 to facilitate mTORC2 activation. A cartoon illustration of ribosome proteins identified as SpCas9 binding proteins in DU145 (**A**) and MDA-MB-231 (**B**) cells. (**C**) IB analysis of WCL from HEK293 cells transfected with increasing doses of GST-PRL26 constructs. Where indicated, cells were serum-starved for 12 h before cell collection. (**D**) IB analysis of WCL from WT or *Sin1^−/−^* MEFs transfected with increasing doses of GST-RPL26. (**E**–**G**, **J**) IB analysis of GST-pulldowns and WCL from HEK293 cells transfected with indicated DNA constructs. Notably, 0.5% Triton X-100 was used in experiments in panels (E) and (F), while 0.5% NP-40 was used in experiments in panel (G). (**H**, **I**) IB analysis of Flag-IPs, HA-IPs, and WCL from HEK293T cells transfected with indicated DNA constructs. (**K**, **L**) IB analysis of WCL from HEK293 cells transfected with increasing doses of GST-RPL23a. Where indicated, cells were serum-starved for 12 h before cell collection. (**M**, **N**) IB analysis of GST-pulldowns and WCL from HEK293 cells transfected with indicated DNA constructs. (**O**) IB analysis of GST-pulldowns and WCL from HEK293 cells transfected with indicated DNA constructs. Where indicated, cells were serum-starved for 12 h before cell collection. (**P**–**R**) Representative *in vitro* mTORC2 kinase assays. IB analysis of mTORC2 kinase reactions. Where indicated, HA-Sin1-expressing HEK293 cells were serum starved for 12 h before HA-IPs to obtain Sin1-containing mTORC2 kinase complexes. (**S**) An *in vitro* HA-Sin1 pulldown assay showing recombinant Cas9 proteins enhance HA-Sin1 IPs binding to recombinant GST-RPL23a proteins. (**T**) A cartoon illustration of the proposed model, where growth factor signaling promotes ribosome binding to mTORC2, facilitating its activation. In the presence of SpCas9, SpCas9 bridges and enhances the ribosome-mTORC2 interaction, bypassing the need for growth factors in ribosome-mediated mTORC2 activation.

Although ribosomal association has been reported to activate mTORC2 [38], the underlying mechanisms remain unclear. SpCas9 interactome analysis in DU145 and MDA-MB-231 cells identified RPL26 as a shared hit (Fig. 4A and B), along with additional ribosomal proteins (Fig. 2E and G). Notably, RPL26 has been previously shown to associate with mTORC2 [38]. To investigate whether RPL26 facilitates mTORC2 activation, we examined its effects on Akt phosphorylation. We found that ectopic RPL26 expression enhanced mTORC2 activation, as evidenced by increased Akt-pS473 levels under both normal and serum-starved conditions (Fig. 4C), in a manner dependent on Sin1 (Fig. 4D). Further interaction studies revealed that RPL26 specifically bound to Sin1, but not to other components of the mTORC2 complex (Fig. 4E). This interaction was primarily mediated by the N-terminal and RBD domains of Sin1 (Fig. 4F), enabling RPL26 to associate with the mTORC2 complex (Fig. 4G).

Previously, we reported that the Sin1-PH domain suppresses mTORC2 kinase activity, and that PI(3,4,5)P3 binding to Sin1-PH exposes the mTOR kinase active site, leading to mTORC2 activation [31]. Here, we observed that RPL26 binding disrupted Sin1-PH suppression of the mTOR kinase domain (Fig. 4H), suggesting a potential mechanism by which ribosomal association promotes mTORC2 activation. Moreover, we found that Cas9 also bound to Sin1 (and GβL), but not other mTOR components in cells (Fig. 4I), via its PH-domain (Supplementary Fig. S4A), and that Cas9 expression enhanced the interaction between RPL26 and Sin1 (Fig. 4J).

In addition to RPL26, we also validated another hit from our established Cas9 interactome, RPL23a, which bound to Cas9 in cells (Supplementary Fig. S4B and C). Similar to RPL26, ectopic expression of RPL23a increased Akt-pS473 signals under both normal (Fig. 4K) and serum starvation conditions (Fig. 4L). RPL23a also interacted with Sin1, but not other mTORC2 subunits (Fig. 4M), and Cas9 significantly enhanced RPL23a association with Sin1 under both normal (Fig. 4N) and serum starvation (Fig. 4O) conditions. Importantly, using *in vitro* mTORC2 kinase assays that we established previously [31, 40], we observed reduced mTORC2 activity when mTORC2 kinase complexes, immunoprecipitated by Sin1, were subjected to serum starvation (Fig. 4P and Supplementary Fig. S4D). The addition of recombinant Cas9 proteins to these *in vitro* mTORC2 kinase reactions enhanced mTORC2 activity (Fig. 4Q and R) because Cas9 enhanced mTORC2 complex interactions with RPL23a (Fig. 4S). Supporting the notion that Cas9 “glues” RPLs to mTORC2 to enhance mTORC2 activation, depletion of endogenous RPL26 (Supplementary Fig. S4E) or RPL23a (Supplementary Fig. S4F) in Cas9-expressing DU145 cells reduced Akt activity. Collectively, these findings suggest that Cas9 may function as a “glue”, enhancing the interaction between ribosomes (such as RPL26 and RPL23a) and Sin1 to facilitate ribosome-mediated mTORC2 activation. Given that growth factor signaling is essential for RPL26’s association with mTORC2 [38], our data also offer an explanation for how Cas9 maintains mTORC2 activation even under serum-starved conditions (Fig. 4T).

## Discussion

Our study examined the effects of stable SpCas9 expression on the growth of 32 cell lines across 9 cancer types and non-cancerous lines using 2D colony formation assays. Both viral infection by NC and SpCas9 expression were found to induce cell growth changes in certain cell lines. However, stable SpCas9 expression affected a subset of but not all cell lines, revealing a context-dependent role of SpCas9 in cell growth control. In this study, we focused on understanding how SpCas9 facilitates cell growth. By establishing the SpCas9 interactome in DU145 and MDA-MB-231 cells, where SpCas9 expression promotes cell growth, ribosome proteins were identified as top, shared binding partners. Further mechanistic investigations showed that ribosomal subunits, such as RPL26 and RPL23a, interact with the Sin1 subunit of the mTORC2 complex. This binding releases Sin1-PH inhibition of the mTOR kinase domain (a mechanism reported previously [31]), allowing for mTORC2 activation. Interestingly, SpCas9 also binds to Sin1, enhancing RPL interactions with Sin1 and promoting ribosome-mediated mTORC2 activation. Previous studies have shown that growth factor signaling promotes ribosome association with mTORC2 for activation [38]. In our study, we found that SpCas9 may act as a “glue” to bridge and enhance binding of ribosomes to mTORC2, even under serum starvation conditions when growth factors are unavailable, thus maintaining mTORC2 activation. This mechanism provides a possible explanation for the cell growth increase induced by SpCas9 expression.

Given that in CRISPR-mediated loss-of-function screens, Cas9 is typically stably introduced into target cells, tissues, or animals, followed by transient transfection of sgRNAs [41], our findings immediately raise concerns about potential bias in CRISPR-mediated screens due to altered cell growth phenotypes. More importantly, our results also highlight safety concerns regarding the use of Cas9 in gene therapy in humans, especially the unwanted potential side effects from sustained Cas9 expression from engineered cells used in these therapies. Our study suggests that our identified Cas9/RPLs/mTORC2 signaling contributes to stable Cas9 expression-altered cell growth, revealing that gaining a deeper understanding of how mammalian cells respond to bacterial Cas9 expression is crucial to address these concerns and ensure the safe use of Cas9-based technologies.

During viral or bacterial infections, it has been reported that pathogens can hijack the host’s mRNA translation machinery, including ribosomes, to ensure efficient translation of viral or bacterial proteins [42–44]. This can be achieved through various mechanisms, such as recruiting host ribosomes, hijacking mRNA translation initiation factors, bypassing 5′-cap requirements using viral RNA [45], or employing viral-encoded proteins to interfere with host mRNA translation. Similar strategies have been observed in bacterial infections, where host ribosome function is modulated to favor bacterial protein production [46]. Given that SpCas9 is a bacterial protein, its influence on host ribosome function and cellular behavior is not entirely unexpected. These findings have broader implications for gene- and base-editing technologies that rely on bacterial proteins, underscoring the need for further investigation into their potential cellular effects. Our study aims to inform the design of new Cas9 variants with minimal unintended cellular impacts, which can be validated through functional assays. Ultimately, we strive to enhance the safety of Cas9-mediated CRISPR genome editing and gene therapy by engineering novel Cas9 variants that mitigate these unwanted side effects.

Our study has several limitations. First, we examined only SpCas9 and did not assess other bacterial Cas9 proteins, leaving it unclear whether and how they might similarly influence cell growth. Second, our focus was limited to cell growth using a 2D assay, without investigating other cellular behaviors such as migration, stress responses, treatment sensitivity, or tumor immunity, which warrant further exploration. Third, while ribosomal components were identified as common hits in our proteomic analyses of DU145 and MDA-MB-231 cells, DU145 cells specifically showed enrichment of mitochondrial RPL/RPS (mRPL/mRPS) proteins. Whether Cas9 affects mitochondrial mRNA translation and whether this contributes to Cas9-induced alterations in cell growth remain open questions for future investigation.

## Supplementary Material

gkaf965_Supplemental_File

## Data Availability

Proteomics data were deposited at PRIDE with the project accession number PXD064062. The RNA-seq data have been deposited in the GEO database under accession number GSE304365. The data underlying this article are available in the article and in its online supplementary material.

## Notes

Present address: Department of Orthopaedics and Traumatology, Li Ka Shing Institute of Health Sciences, Chinese University of Hong Kong, Hong Kong SAR 999077, China

## References

[1] Mali P, Yang L, Esvelt KM et al. RNA-guided human genome engineering via Cas9. Science. 2013; 339:823–6. 10.1126/science.1232033. 23287722 PMC3712628

[2] Cong L, Ran FA, Cox D et al. Multiplex genome engineering using CRISPR/Cas systems. Science. 2013; 339:819–23. 10.1126/science.1231143. 23287718 PMC3795411

[3] Chen S, Sanjana NE, Zheng K et al. Genome-wide CRISPR screen in a mouse model of tumor growth and metastasis. Cell. 2015; 160:1246–60. 10.1016/j.cell.2015.02.038. 25748654 PMC4380877

[4] Korkmaz G, Lopes R, Ugalde AP et al. Functional genetic screens for enhancer elements in the human genome using CRISPR–Cas9. Nat Biotechnol. 2016; 34:192–8. 10.1038/nbt.3450. 26751173

[5] Huang A, Garraway LA, Ashworth A et al. Synthetic lethality as an engine for cancer drug target discovery. Nat Rev Drug Discov. 2020; 19:23–38. 10.1038/s41573-019-0046-z. 31712683

[6] Kurata M, Yamamoto K, Moriarity BS et al. CRISPR/Cas9 library screening for drug target discovery. J Hum Genet. 2018; 63:179–86. 10.1038/s10038-017-0376-9. 29158600

[7] Yin H, Xue W, Chen S et al. Genome editing with Cas9 in adult mice corrects a disease mutation and phenotype. Nat Biotechnol. 2014; 32:551–3. 10.1038/nbt.2884. 24681508 PMC4157757

[8] Nelson CE, Hakim CH, Ousterout DG et al. *In vivo* genome editing improves muscle function in a mouse model of Duchenne muscular dystrophy. Science. 2016; 351:403–7. 10.1126/science.aad5143. 26721684 PMC4883596

[9] Long C, Amoasii L, Mireault AA et al. Postnatal genome editing partially restores dystrophin expression in a mouse model of muscular dystrophy. Science. 2016; 351:400–3. 10.1126/science.aad5725. 26721683 PMC4760628

[10] Tabebordbar M, Zhu K, Cheng JK et al. *In vivo* gene editing in dystrophic mouse muscle and muscle stem cells. Science. 2016; 351:407–11. 10.1126/science.aad5177. 26721686 PMC4924477

[11] Amoasii L, Hildyard JCW, Li H et al. Gene editing restores dystrophin expression in a canine model of duchenne muscular dystrophy. Science. 2018; 362:86–91. 10.1126/science.aau1549. 30166439 PMC6205228

[12] Xiao-Jie L, Hui-Ying X, Zun-Ping K et al. CRISPR–Cas9: a new and promising player in gene therapy. J Med Genet. 2015; 52:289–96. 10.1136/jmedgenet-2014-102968. 25713109

[13] Gaudelli NM, Komor AC, Rees HA et al. Programmable base editing of A*T to G*C in genomic DNA without DNA cleavage. Nature. 2017; 551:464–71. 10.1038/nature24644. 29160308 PMC5726555

[14] Jeong YK, Song B, Bae S Current status and challenges of DNA base editing tools. Mol Ther. 2020; 28:1938–52. 10.1016/j.ymthe.2020.07.021. 32763143 PMC7474268

[15] Carsten Trevor Charlesworth PSD, Dever DP, Dejene B et al. Identification of preexisting adaptive immunity to Cas9 proteins in humans. Nat Med. 2019; 25:249–54. 10.1101/243345. 30692695 PMC7199589

[16] Platt RJ, Chen S, Zhou Y et al. CRISPR–Cas9 knockin mice for genome editing and cancer modeling. Cell. 2014; 159:440–55. 10.1016/j.cell.2014.09.014. 25263330 PMC4265475

[17] Haapaniemi E, Botla S, Persson J et al. CRISPR–Cas9 genome editing induces a p53-mediated DNA damage response. Nat Med. 2018; 24:927–30. 10.1038/s41591-018-0049-z. 29892067

[18] Ihry RJ, Worringer KA, Salick MR et al. p53 inhibits CRISPR–Cas9 engineering in human pluripotent stem cells. Nat Med. 2018; 24:939–46. 10.1038/s41591-018-0050-6. 29892062

[19] Kosicki M, Tomberg K, Bradley A Repair of double-strand breaks induced by CRISPR–Cas9 leads to large deletions and complex rearrangements. Nat Biotechnol. 2018; 36:765–71. 10.1038/nbt.4192. 30010673 PMC6390938

[20] Tsuchida CA, Brandes N, Bueno R et al. Mitigation of chromosome loss in clinical CRISPR–Cas9-engineered T cells. Cell. 2023; 186:4567–82. 10.1016/j.cell.2023.08.041. 37794590 PMC10664023

[21] Tao J, Wang Q, Mendez-Dorantes C et al. Frequency and mechanisms of LINE-1 retrotransposon insertions at CRISPR/Cas9 sites. Nat Commun. 2022; 13:3685 10.1038/s41467-022-31322-3. 35760782 PMC9237045

[22] Fiumara M, Ferrari S, Omer-Javed A et al. Genotoxic effects of base and prime editing in human hematopoietic stem cells. Nat Biotechnol. 2023; 42:877–91. 37679541 10.1038/s41587-023-01915-4 PMC11180610

[23] Chen JS, Ma E, Harrington LB et al. CRISPR-Cas12a target binding unleashes indiscriminate single-stranded DNase activity. Science. 2018; 360:436–9. 10.1126/science.aar6245. 29449511 PMC6628903

[24] Liu JJ, Orlova N, Oakes BL et al. CasX enzymes comprise a distinct family of RNA-guided genome editors. Nature. 2019; 566:218–23. 10.1038/s41586-019-0908-x. 30718774 PMC6662743

[25] Burstein D, Harrington LB, Strutt SC et al. New CRISPR–Cas systems from uncultivated microbes. Nature. 2017; 542:237–41. 10.1038/nature21059. 28005056 PMC5300952

[26] Saito M, Xu P, Faure G et al. Fanzor is a eukaryotic programmable RNA-guided endonuclease. Nature. 2023; 620:660–8. 10.1038/s41586-023-06356-2. 37380027 PMC10432273

[27] Yu L, Deng Y, Wang X et al. Co-targeting JAK1/STAT6/GAS6/TAM signaling improves chemotherapy efficacy in Ewing sarcoma. Nat Commun. 2024; 15:5292 10.1038/s41467-024-49667-2. 38906855 PMC11192891

[28] Deng Y, Hahn Q, Yu L et al. 2'3'-cGAMP interactome identifies 2'3'-cGAMP/Rab18/FosB signaling in cell migration control independent of innate immunity. Sci Adv. 2024; 10:eado7024 10.1126/sciadv.ado7024. 39413198 PMC11482326

[29] Chen J, Su S, Pickar-Oliver A et al. Engineered Cas9 variants bypass Keap1-mediated degradation in human cells and enhance epigenome editing efficiency. Nucleic Acids Res. 2024; 52:11536–51. 10.1093/nar/gkae761. 39228373 PMC11514467

[30] Zhu Z, Zhou X, Du H et al. STING suppresses mitochondrial VDAC2 to govern RCC growth independent of innate immunity. Adv Sci. 2023; 10:e2203718 10.1002/advs.202203718. PMC9875608 36445063

[31] Liu P, Gan W, Chin YR et al. PtdIns(3,4,5)P3-dependent activation of the mTORC2 kinase complex. Cancer Discov. 2015; 5:1194–209. 10.1158/2159-8290.CD-15-0460. 26293922 PMC4631654

[32] Su S, Chen J, Jiang Y et al. SPOP and OTUD7A control EWS-FLI1 protein stability to govern ewing sarcoma growth. Adv Sci. 2021; 8:e2004846 10.1002/advs.202004846. PMC8292909 34060252

[33] Cox J, Neuhauser N, Michalski A et al. Andromeda: a peptide search engine integrated into the MaxQuant environment. J Proteome Res. 2011; 10:1794–805. 10.1021/pr101065j. 21254760

[34] Cox J, Mann M MaxQuant enables high peptide identification rates, individualized p.p.b.-range mass accuracies and proteome-wide protein quantification. Nat Biotechnol. 2008; 26:1367–72. 10.1038/nbt.1511. 19029910

[35] Perez-Riverol Y, Bai J, Bandla C et al. The PRIDE database resources in 2022: a hub for mass spectrometry-based proteomics evidences. Nucleic Acids Res. 2022; 50:D543–52. 10.1093/nar/gkab1038. 34723319 PMC8728295

[36] Ran FA, Hsu PD, Wright J et al. Genome engineering using the CRISPR–Cas9 system. Nat Protoc. 2013; 8:2281–308. 10.1038/nprot.2013.143. 24157548 PMC3969860

[37] Major RM, Mills CA, Xing L et al. Exploring the cytoplasmic retention of CRISPR–Cas9 in eukaryotic cells: the role of nuclear localization signals and ribosomal interactions. CRISPR J. 2025; 8:120–36. 10.1089/crispr.2024.0074. 40019800

[38] Zinzalla V, Stracka D, Oppliger W et al. Activation of mTORC2 by association with the ribosome. Cell. 2011; 144:757–68. 10.1016/j.cell.2011.02.014. 21376236

[39] Fu W, Hall MN Regulation of mTORC2 signaling. Genes. 2020; 11:1045 10.3390/genes11091045. 32899613 PMC7564249

[40] Liu P, Begley M, Michowski W et al. Cell-cycle-regulated activation of akt kinase by phosphorylation at its carboxyl terminus. Nature. 2014; 508:541–5. 10.1038/nature13079. 24670654 PMC4076493

[41] Bock C, Datlinger P, Chardon F et al. High-content CRISPR screening. Nat Rev Methods Primers. 2022; 2:9 10.1038/s43586-021-00093-4. 37214176 PMC10200264

[42] Walsh D, Mohr I Viral subversion of the host protein synthesis machinery. Nat Rev Microbiol. 2011; 9:860–75. 10.1038/nrmicro2655. 22002165 PMC7097311

[43] Stern-Ginossar N, Thompson SR, Mathews MB et al. Translational control in virus-infected cells. Cold Spring Harb Perspect Biol. 2019; 11:a033001 10.1101/cshperspect.a033001. 29891561 PMC6396331

[44] Rozman B, Fisher T, Stern-Ginossar N Translation—a tug of war during viral infection. Mol Cell. 2023; 83:481–95. 10.1016/j.molcel.2022.10.012. 36334591

[45] Lopez-Ulloa B, Fuentes Y, Pizarro-Ortega MS et al. RNA-binding proteins as regulators of internal initiation of viral mRNA translation. Viruses. 2022; 14:188 10.3390/v14020188. 35215780 PMC8879377

[46] Mohr I, Sonenberg N Host translation at the nexus of infection and immunity. Cell Host Microbe. 2012; 12:470–83. 10.1016/j.chom.2012.09.006. 23084916 PMC7104986

