# Supplementary material for: Stable Cas9 expression regulates cell growth by facilitating mTORC2 activation
Source: Nucleic Acids Res. 2025 Sep 29;53(18):gkaf965. doi: 10.1093/nar/gkaf965 (PMC12477591; doi:10.1093/nar/gkaf965)
Supplement: gkaf965_Supplemental_File [file gkaf965_supplemental_file.pdf]

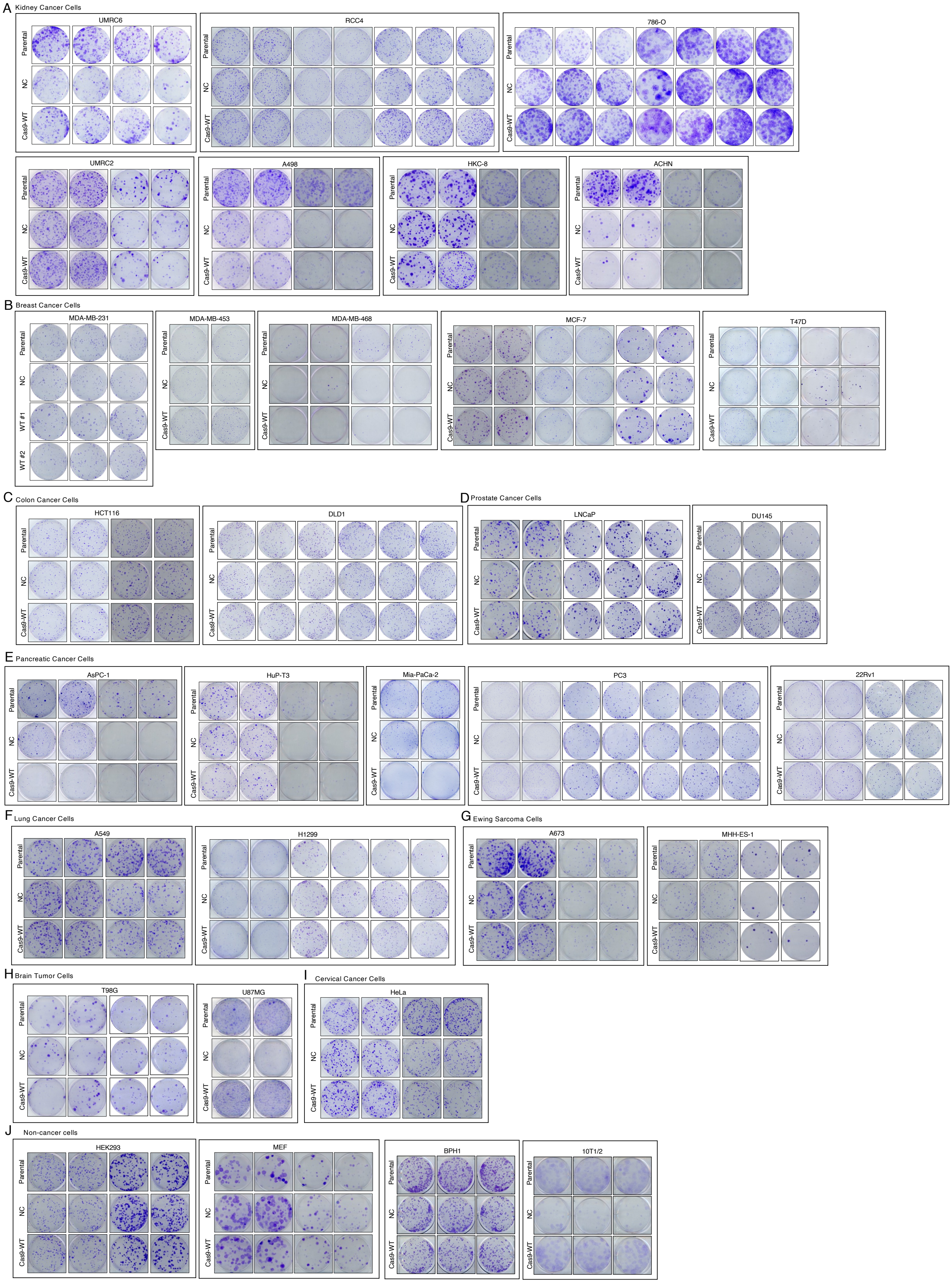
A Kidney Cancer Cells
UMRC6
RCC4
786-O
UMRC2
A498
HKC-8
ACHN
Parental
NC
Cas9-WT
B Breast Cancer Cells
MDA-MB-231
MDA-MB-453
MDA-MB-468
MCF-7
T47D
Parental
NC
WT #1
WT #2
Cas9-WT
C Colon Cancer Cells
HCT116
DLD1
D Prostate Cancer Cells
LNCaP
DU145
E Pancreatic Cancer Cells
AsPC-1
HuP-T3
Mia-PaCa-2
PC3
22Rv1
F Lung Cancer Cells
A549
H1299
G Ewing Sarcoma Cells
A673
MHH-ES-1
H Brain Tumor Cells
T98G
U87MG
I Cervical Cancer Cells
HeLa
J Non-cancer cells
HEK293
MEF
BPH1
10T1/2

Figure S1. Profiling effects of stable SpCas9 expression on growth of 32 cell lines as indicated.
(A-J) Representative images of 2D colony formation assays using indicated cells.

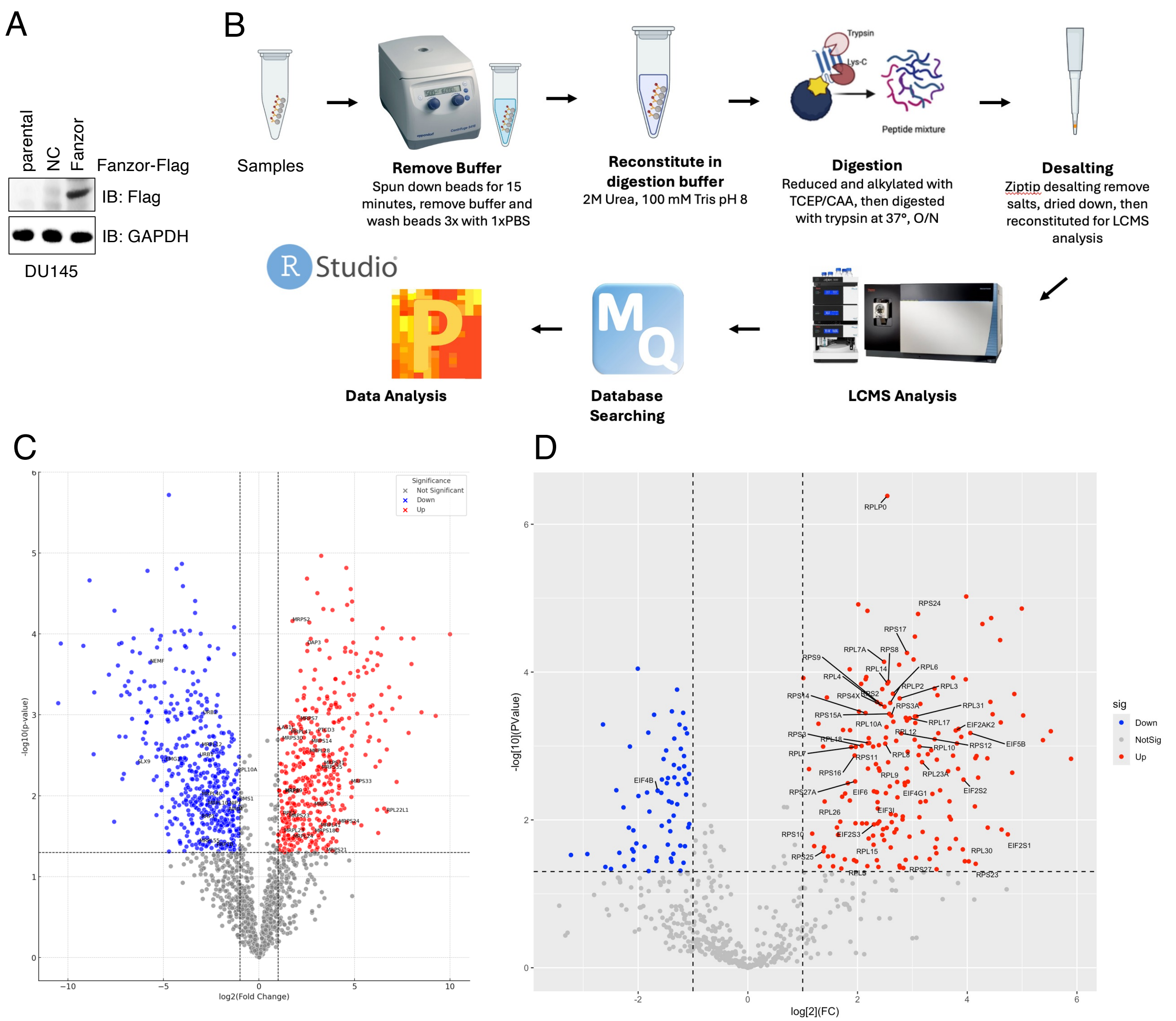

Figure S2. SpCas9 interactome in DU145 and MDA-MB-231 cells identifies ribosome proteins.

(A) IB analysis of WCL from indicated DU145 cells. (B) A cartoon illustration of the procedures for mass spectrometry analysis of SpCas9-Flag-IP samples from DU145 or MDA-MB-231 cells. (C, D) Volcano plots revealing identified SpCas9 binding proteins in DU145 (B) and MDA-MB-231 (C) cells.

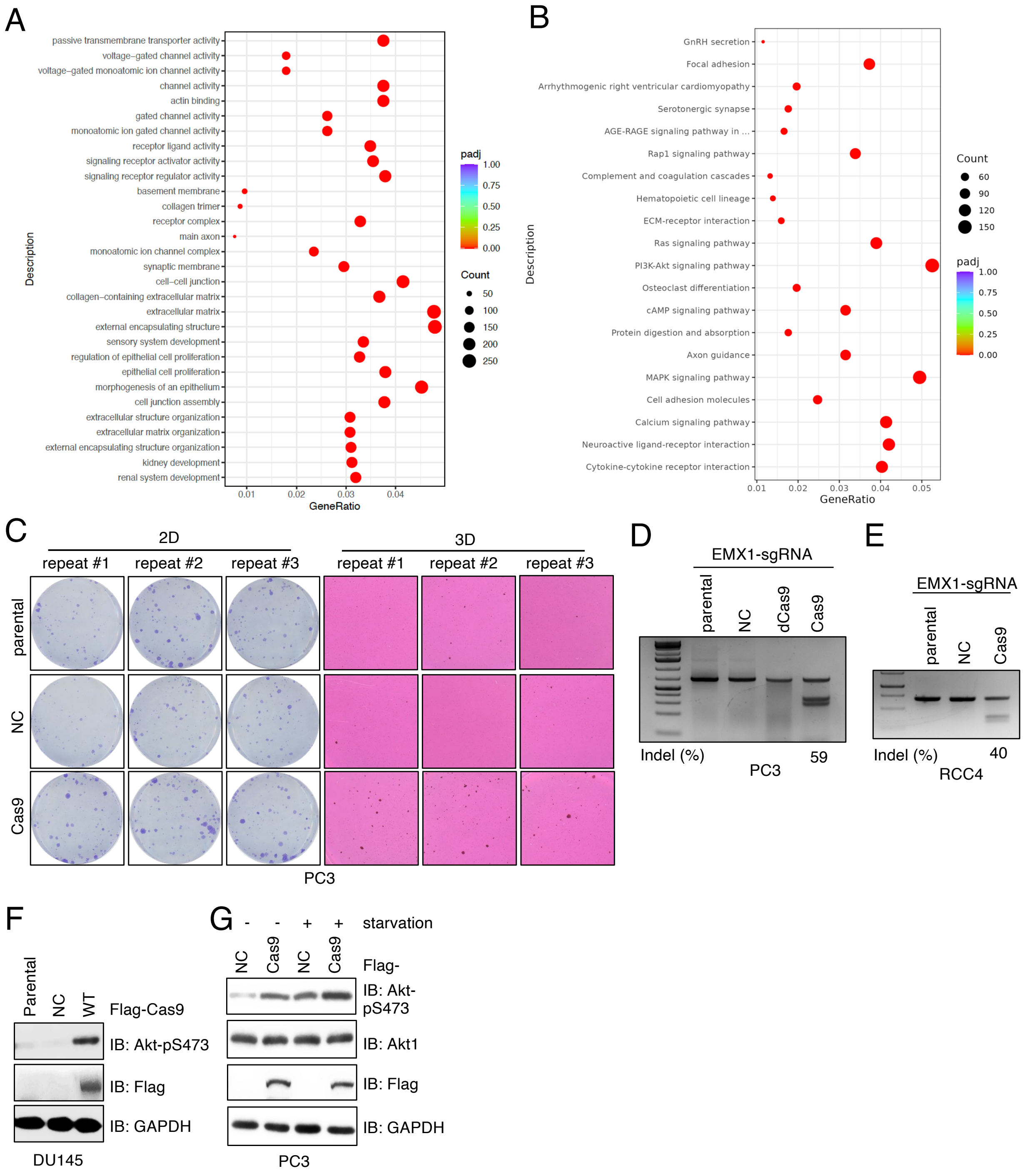

Figure S3. Stable SpCas9 expression facilitates mTORC2/Akt activation in cells. (A, B) Representative Gene Ontology (GO) and KEGG pathway analyses of Cas9-induced transcriptomic changes in DU145 cells stably expressing Cas9-WT compared with NC. (C) Representative 2D colony formation assays and 3D soft-agar growth assays of PC3 cells. (D, E) Representative T7E1 assay results from indicated cells transfected with EMX1-sgRNA for 3 days. (F) IB analysis of WCL from indicated DU145 cells. (G) IB analysis of WCL from indicated PC3 cells. Where indicated, cells were serum starved for 12 hours before cell collection.

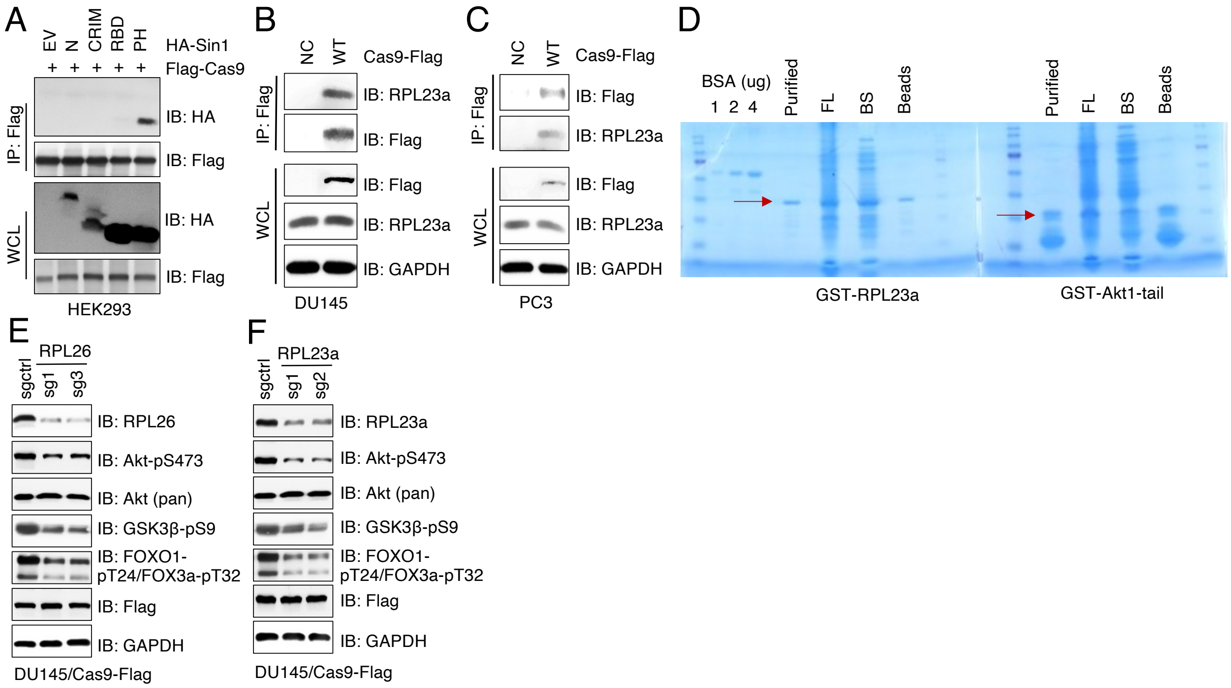

Figure S4. SpCas9 facilitates RPL23a binding to mTORC2.
(A) IB analyses of Flag-IPs and WCL from HEK293 cells transfected with indicated DNA constructs. (B, C) IB analyses of Flag-IPs and WCL from indicated DU145 (A) or PC3 (B) cells. (D) A representative Coomassie blue stained gel for purified recombinant GST-RPL23a and GST-Akt1-tail proteins. (E, F) IB analyses of WCL from indicated DU145 cells stably expressing Cas9 depleted of endogenous RPL26 (E) or RPL23a (F) by sgRNAs.

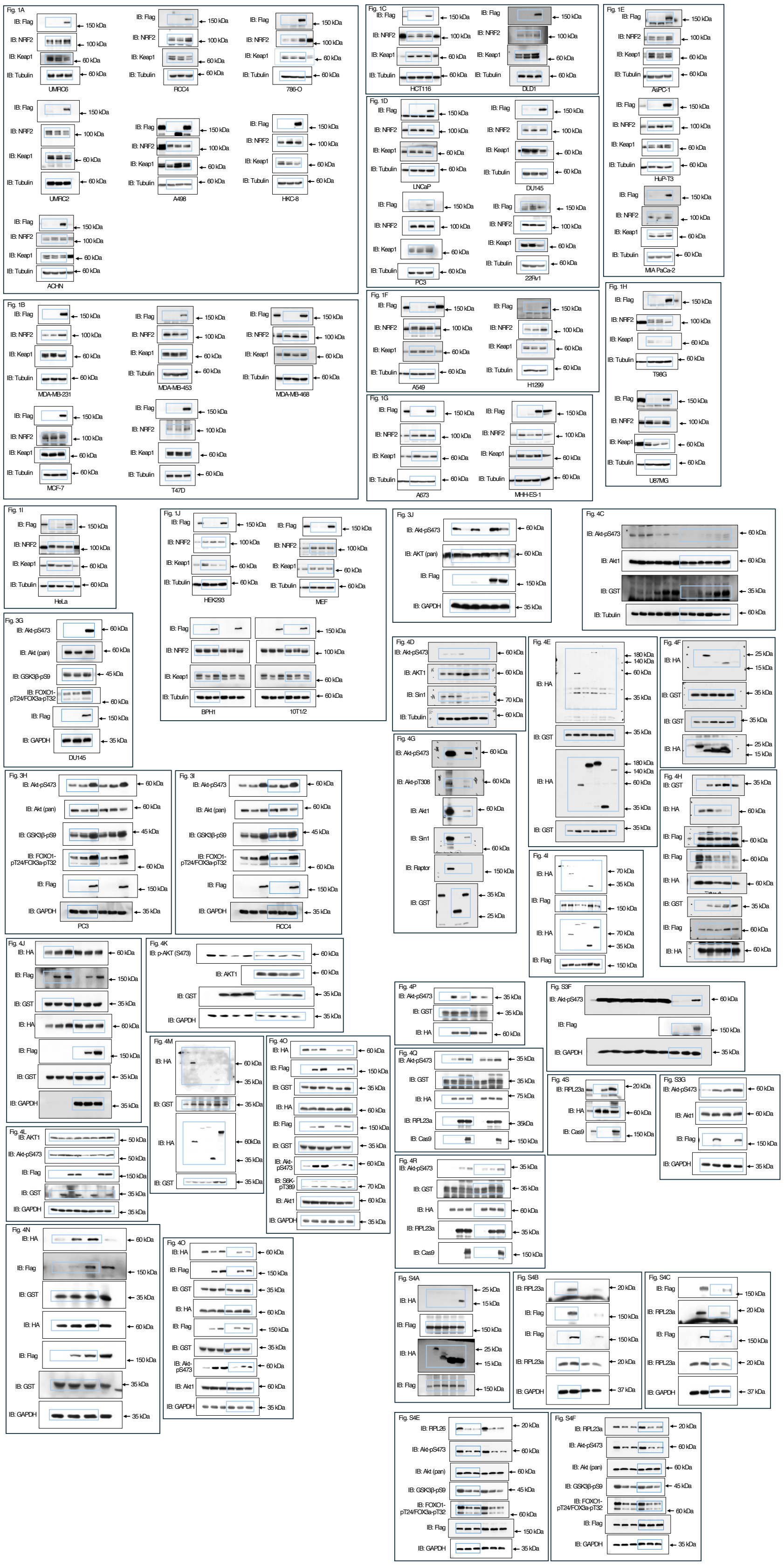

Figure S5. Uncropped, unprocessed western blot images.
